# Supplementary material for: Interaction between Illite and a Pseudomonas stutzeri-Heavy Oil Biodegradation Complex
Source: Microorganisms. 2023 Jan 28;11(2):330. doi: 10.3390/microorganisms11020330 (PMC9960338; doi:10.3390/microorganisms11020330)
Supplement: Supplementary file 1 [file microorganisms-11-00330-s001.zip › microorganisms-2095878-supplementary.pdf]

Supplemental Materials

Interaction between Illite and a *Pseudomonas stutzeri*-Heavy Oil Biodegradation Complex

Lei Li, Yun Yang Wan *, Hong Mei Mu, Sheng Bao Shi and Jian Fa Chen

State Key Laboratory of Petroleum Resources and Prospecting, Research Centre for Geomicrobial Resources and Application, Unconventional Petroleum Research Institute, College of Geosciences, China University of Petroleum, Beijing 102249, China

* Correspondence: wanyunyang@cup.edu.cn

Table S1. The pH, σ and Eh of MVN and illite-*Pst*HO.

Sample	pH	Error	σ(μS/cm)	Error	Eh(mV)	Error
MVN	8.15	±0.12	2529.0	±215.0	165.9	±7.6
P0I0	8.15	±0.09	2550.0	±137.9	171.4	±10.7
P0I8	8.14	±0.11	2435.0	±39.8	159.0	±11.6
P2I0	7.51	±0.13	844.1	±7.7	108.4	±7.2
P2I8	7.66	±0.09	879.0	±24.3	115.8	±7.4
P2I32	7.69	±0.08	893.0	±29.0	123.0	±9.0

Note: See Table 1 for σ, Eh, MVN, and *Pst*HO; see Table 2 for P0I0, P0I8, P2I0, P2I8, and P2I32.

Table S2. Saturated hydrocarbons in heavy oil L1YJC23 and illite-*Pst*HO detected by GC-MS

No.	Compound name	No.	Compound name	No.	Compound name
1	C_{15}-bicyclic sesquiterpene	23	13β(H),14α(H)-C_{29} tricyclic terpane(S)	45	C_{21}-5α(H)-pregnane
2	C_{15}-bicyclic sesquiterpene	24	18α(H)-22,29,30-trisnorneohopane(Ts)	46	C_{22}-5α(H)-homopregnane
3	8β(H)-drimane	25	17α(H)-22,29,30-trisnorhopane(Tm)	47	13β(H),17α(H)-C_{27} diastrane(20S)
4	C_{15}-bicyclic sesquiterpene	26	17α,21β(H)-30 norhopane(C_{29}H)	48	13β(H),17α(H)-C_{27} diastrane(20R)
5	C_{16}-bicyclic sesquiterpene	27	18α,21β(H)-30 norneohopane(C_{29}Ts)	49	13α(H),17β(H)-C_{27} diastrane(20S)
6	C_{15}-bicyclic sesquiterpene	28	C_{30} diahopane	50	13α(H),17β(H)-C_{27} diastrane(20R)
7	C_{16}-bicyclic sesquiterpene	29	17β(H), 21α(H)-30 normoretane	51	5α(H),14α(H),17α(H)-sterane(20S)
8	8β(H)-homodrimane	30	18α(H)-oleanane	52	5α(H),14β(H),17β(H)-sterane(20R)
9	13β(H),14α(H)-C_{19} tricyclic terpane	31	17α(H),21β(H)-hopane(C_{30}H)	53	5α(H),14β(H),17β(H)-sterane(20S)
10	13β(H),14α(H)-C_{20} tricyclic terpane	32	17β(H),21α(H)-moretane(C_{30}M)	54	5α(H),14α(H),17α(H)-sterane(20R)
11	13β(H),14α(H)-C_{21} tricyclic terpane	33	17α(H),21β(H)-30 homohopane(22S)	55	13β(H),17α(H)-C_{29}diastrane(20R)
12	13β(H),14α(H)-C_{22} tricyclic terpane	34	17α(H),21β(H)-30 homohopane(22R)	56	13α(H),17β(H)-C_{29}diastrane(20S)
13	13β(H),14α(H)-C_{23} tricyclic terpane	35	gammacerane	57	24-methyl-5α(H),14α(H),17α(H)-sterane(20S)
14	13β(H),14α(H)-C_{24} tricyclic terpane	36	17β(H),21α(H)-30 homomoretane(22S+22R)	58	24-methyl-5α(H),14β(H),17β(H)-sterane(20R)
15	13β(H),14α(H)-C_{25} tricyclic terpane(R)	37	17α(H),21β(H)-30,31 dihomohopane(22S)	59	24-methyl-5α(H),14β(H),17β(H)-sterane(20S)
16	13β(H),14α(H)-C_{25} tricyclic terpane(S)	38	17α(H),21β(H)-30,31 dihomohopane(22R)	60	24-methyl-5α(H),14α(H),17α(H)-sterane(20R)
17	13β(H),14α(H)-C_{26} tricyclic terpane(R)	39	17α(H),21β(H)-30,31,32 trihomohopane(22S)	61	24-ethyl-5α(H),14α(H),17α(H)-sterane(20S)
18	C_{24} tetracyclic terpane	40	17α(H), 21β(H)-30,31,32trihomohopane(22R)	62	24-ethyl-5α(H),14β(H),17β(H)-sterane(20R)
19	13β(H),14α(H)-C_{26} tricyclic terpane(S)	41	17α(H),21β(H)-30,31,32,33 tetrahomohopane(22S)	63	24-ethyl-5α(H),14β(H),17β(H)-sterane(20S)
20	13β(H),14α(H)-C_{28} tricyclic terpane(R)	42	17α(H),21β(H)-30,31,32,33 tetrahomohopane(22R)	64	24-ethyl-5α(H),14α(H),17α(H)-sterane(20R)
21	13β(H),14α(H)-C_{28} tricyclic terpane(S)	43	17α(H),21β(H)-30,31,32,33,34 pentahomohopane (22S)		
22	13β(H),14α(H)-C_{29} tricyclic terpane(R)	44	17α(H),21β(H)-30,31,32,33,34 pentahomohopane (22R)		

Table S3. Aromatic hydrocarbons in heavy oil L1YJC23 and illite-*Pst*HO detected by GC-MS

No.	Category	Type	Compound name	Abbreviation
1	naphthalene series	Nap	naphthalene	Nap
2		monomethyl naphthalene /MeNap	2-methyl naphthalene	2-MeNap
3			1-methyl naphthalene	1-MeNap
4		dimethyl naphthalene /DMN	1,6-dimethyl naphthalene	1,6-DMN
5			1,5-dimethyl naphthalene	1,5-DMN
6			1,2-dimethyl naphthalene	1,2-DMN
7		trimethyl naphthalene /TMN	1,3,7-trimethyl naphthalene	1,3,7-TMN
8			1,3,6-trimethyl naphthalene	1,3,6-TMN
9			2,3,6-trimethyl naphthalene	2,3,6-TMN
10			1,2,6-trimethyl naphthalene	1,2,6-TMN
11			1,2,4-trimethyl naphthalene	1,2,4-TMN
12			1,2,5-trimethyl naphthalene	1,2,5-TMN
13			1,4,5-trimethyl naphthalene	1,4,5-TMN
14		tetramethyl naphthalene /TeMN	1,3,5,7-tetramethyl naphthalene	1,3,5,7-TeMN
15			1,3,6,7-tetramethyl naphthalene	1,3,6,7-TeMN
16			2,3,6,7-tetramethyl naphthalene	2,3,6,7-TeMN
17			1,2,6,7-tetramethyl naphthalene	1,2,6,7-TeMN
17			1,2,3,7-tetramethyl naphthalene	1,2,3,7-TeMN
19			1,2,3,6-tetramethyl naphthalene	1,2,3,6-TeMN
20		pentamethyl naphthalene /PMN	1,2,4,6,7-pentamethyl naphthalene	1,2,4,6,7-PMN
21			1,2,3,5,7-pentamethyl naphthalene	1,2,3,5,7-PMN
22			1,2,3,6,7-pentamethyl naphthalene	1,2,3,6,7-PMN
23			1,2,3,5,6-pentamethyl naphthalene	1,2,3,5,6-PMN
24	phenanthrene series	Phe	phenanthrene	Phe
25		monomethyl phenanthrene /MePhe	3-methyl phenanthrene	3-MePhe
26			2-methyl phenanthrene	2-MePhe
27			9-methyl phenanthrene	9-MePhe
28			1-methyl phenanthrene	1-MePhe
29		monoethyl phenanthrene	3-ethyl phenanthrene	3-EPhe

30		/EtPhe	1-ethyl phenanthrene	1-EPhe
31		dimethyl phenanthrene /DMP	1,7-dimethyl phenanthrene	1,7-DMP
32			2,3-dimethyl phenanthrene	2,3-DMP
33			1,8-dimethyl phenanthrene	1,8-DMP
34			1,2-dimethyl phenanthrene	1,2-DMP
35		trimethyl phenanthrene /TMP	1,3,8-trimethyl phenanthrene	1,3,8-TMP
36			2,3,10-trimethyl phenanthrene	2,3,10-TMP
37			1,6,7-trimethyl phenanthrene	1,6,7-TMP
38			1,2,6-trimethyl phenanthrene	1,2,6-TMP
39			1,2,8-trimethyl phenanthrene	1,2,8-TMP
40	fluorene series	Fle	fluorene	Fle
41		monomethyl fluorene /MeFle	3-methyl fluorene	3-MeFle
42			2-methyl fluorene	2-MeFle
43			1-methyl fluorene	1-MeFle
44			4-methyl fluorene	4-MeFle
45		DBT	dibenzothiophene	DBT
46		monomethyl dibenzothiophene /MDBT	4-methyl dibenzothiophene	4-MDBT
47			1-methyl dibenzothiophene	1-MDBT
48		monoethyl dibenzothiophene/EDBT	4-ethyl dibenzothiophene	4-EDBT
49		dimethyl dibenzothiophene /DMDBT	4,6-dimethyl dibenzothiophene	4,6-DMDBT
50			2,4-dimethyl dibenzothiophene	2,4-DMDBT
51			2,6-dimethyl dibenzothiophene	2,6-DMDBT
52			3,6-dimethyl dibenzothiophene	3,6-DMDBT
53			2,8-dimethyl dibenzothiophene	2,8-DMDBT
54			1,7-dimethyl dibenzothiophene	1,7-DMDBT
55			1,2-dimethyl dibenzothiophene	1,2-DMDBT
56		DBF	dibenzofuran	DBF
57		monomethyl dibenzofuran /MDBF	4-methyl dibenzofuran	4-MDBF
58			1-methyl dibenzofuran	1-MDBF
59	biphenyl series	Bph	biphenyl	Bph
60		monomethyl biphenyl	3-methyl biphenyl	3-MeBph

61		/MeBph	4-methyl biphenyl	4-MeBph
62		EtBph	3-ethyl biphenyl	3-EtBph
63		dimethyl biphenyl /DMB	3,5-dimethyl biphenyl	3,5-DMB
64			3,3'-dimethyl biphenyl	3,3'-DMB
65			3,4'-dimethyl biphenyl	3,4'-DMB
66			4,4'-dimethyl biphenyl	4,4'-DMB
67			3,4-dimethyl biphenyl	3,4-DMB
68		trimethyl biphenyl /TMB	3,5,3'-trimethyl biphenyl	3,5,3'-TMB
69			3,5,4'-trimethyl biphenyl	3,5,4'-TMB
70			3,4,3'-trimethyl biphenyl	3,4,3'-TMB
71			3,4,4'-trimethyl biphenyl	3,4,4'-TMB
72	high-ring number (≥4) aromatic hydrocarbons /HRAHs		fluoranthene	Fla
73			pyrene	Pyr
74			benz(a)fluorene	B(a)Fle
75			Benz(b)fluorene	B(b)Fle
76		monomethyl pyrene /MePyr	2-methyl pyrene	2-Mepyr
77			4-methyl pyrene	4-Mepyr
78			1-methyl pyrene	1-Mepyr
79			Benzo(a)anthracene	B(a)A
80			chrysene	Chr
81		monomethyl chrysene /MeChr	3-methyl chrysene	3-MeChr
82			2-methyl chrysene	2-MeChr
83			4-methyl chrysene	4-MeChr
84			6-methyl chrysene	6-MeChr
85			1-methyl chrysene	1-MeChr
86			Benzo(b)fluoranthene	B(b)F
87			Benzo(k)fluoranthene	B(k)F
88			Benzo(e)pyrene	B(e)P
89			Benzo(a)pyrene	B(a)P
90			perylene	Per
91		triaromatic steroid	C20 triaromatic steroid	C20 TAS

92	/TAS	C21 triaromatic steroid	C21 TAS
93		C26 triaromatic steroid(20S)	C26 TAS(20S)
94		C28 triaromatic steroid(20S)	C28 TAS(20S)
95		C27 triaromatic steroid(20R)	C27 TAS(20R)
96		C28 triaromatic steroid(20R)	C28 TAS(20R)

Note: This table does not contain co overflow compounds

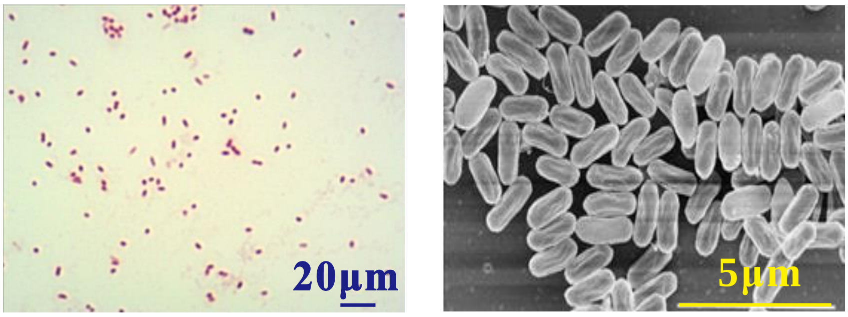

(a) (b)

Figure S1. Micrographs of cells of *Pseudomonas stutzeri* strain L1SHX-3X in an Eclipse Ni-U upright microscope (Nikon, Tokyo, Japan) (**a**) and a VEGA 3 SEM (TESCAN, Brno, Czech Republic) (**b**) after 6 days of reactivation by MVN-R from freeze-dried powder (storage at −80 °C) in the shaker with 120 rpm at 35 °C

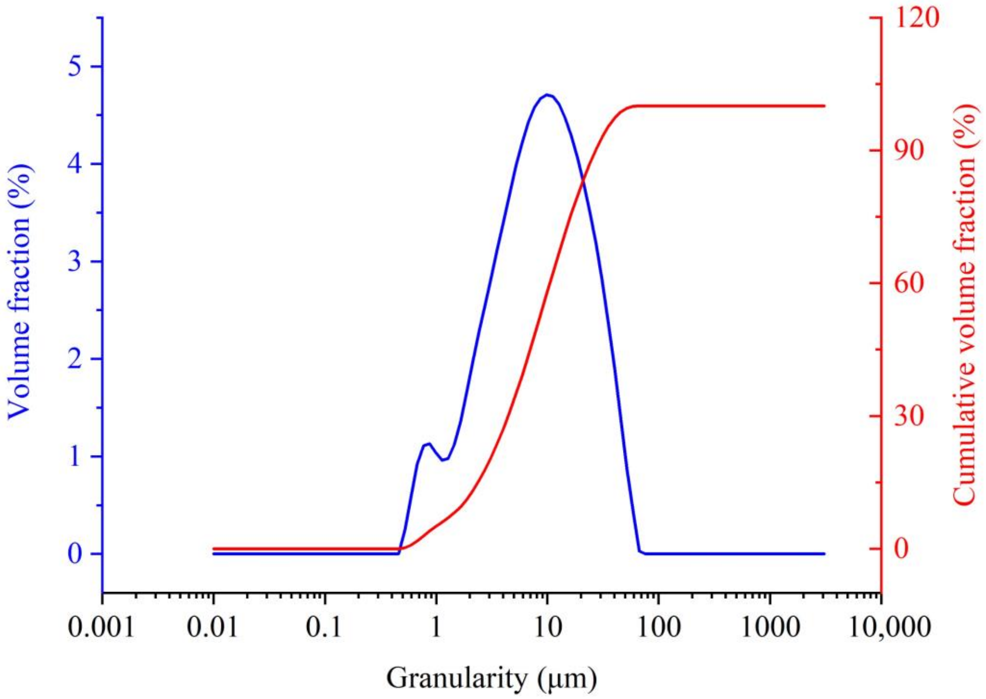

Figure S2. Particle size distribution of illite measured by Mastersizer 3000 (Malvern Panalytical, Great Malvern, UK)

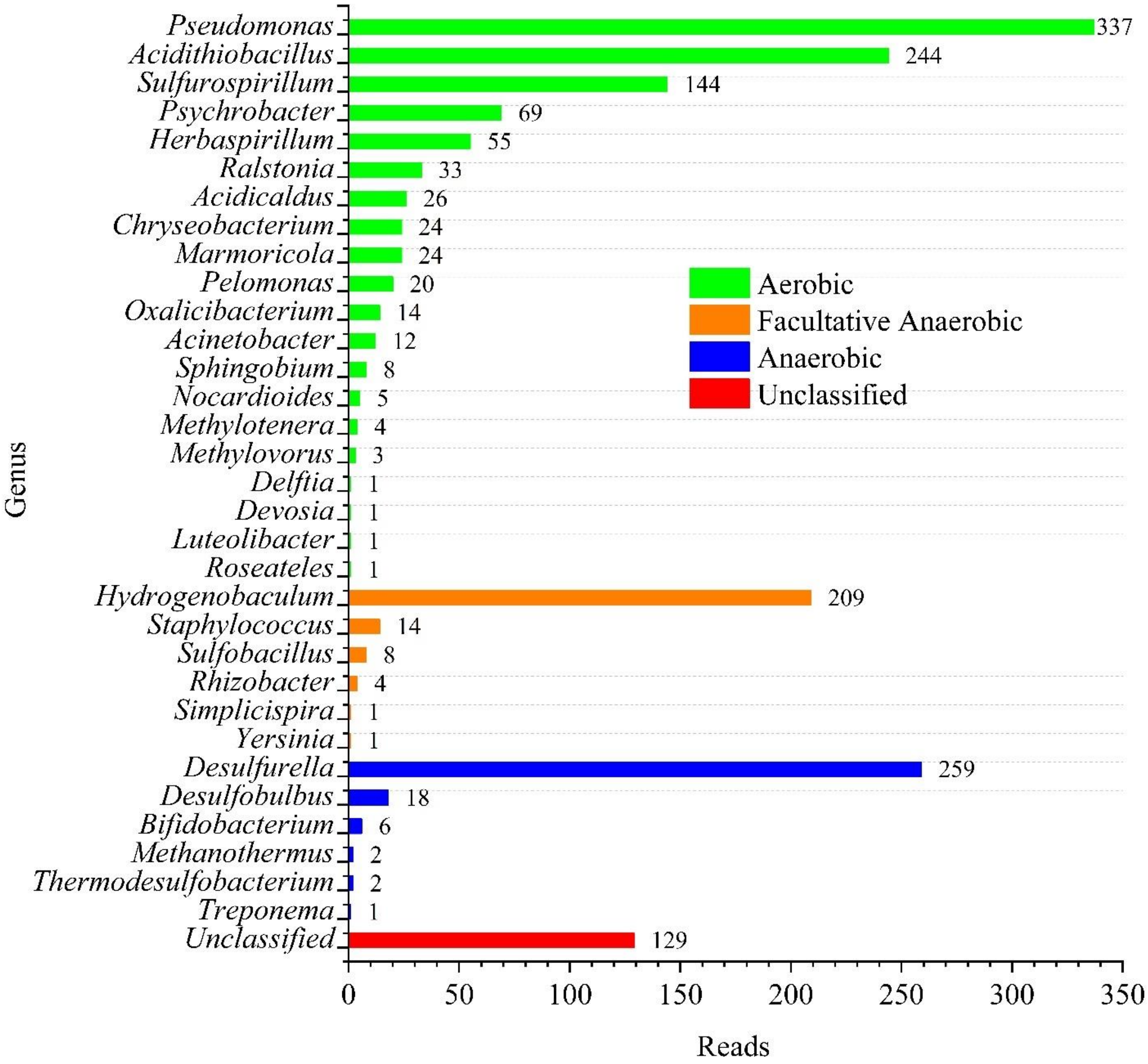

Figure S3. Genus-level analysis of *in situ* microorganisms in heavy oil L1YJC23

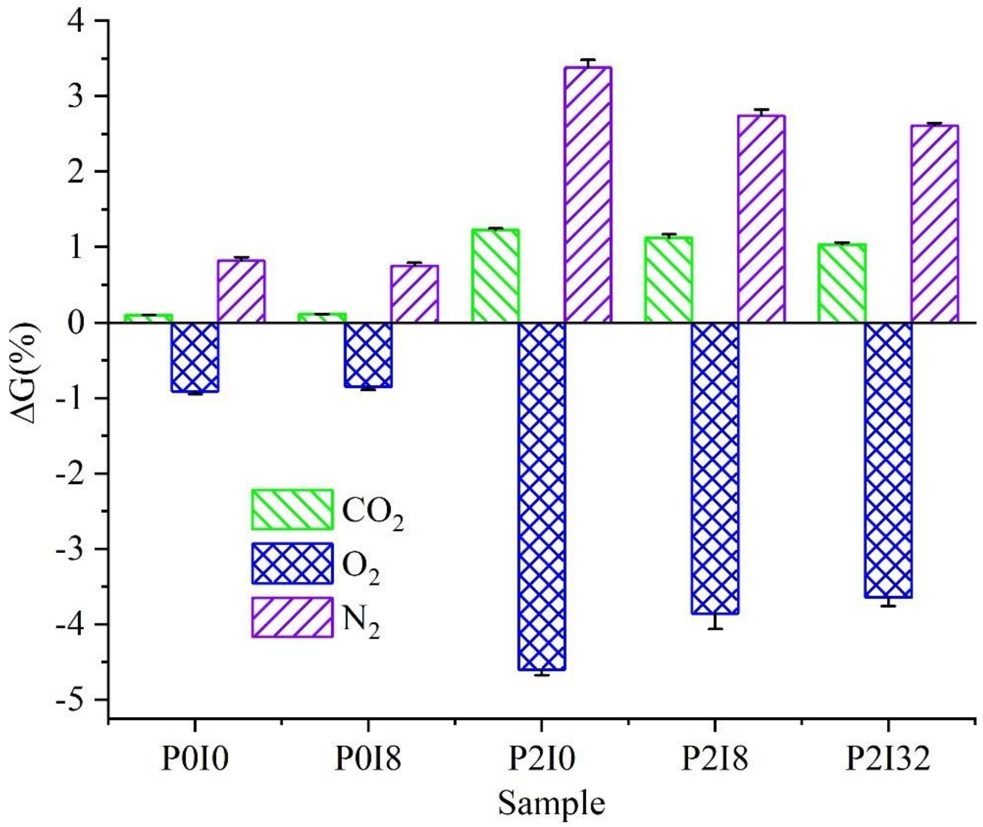

Figure S4. Difference in CO_2, O_2, and N_2 contents between illite-*Pst*HO and atmosphere
Note: See Table 1 for ΔG; see Table 2 for P0I0, P0I8, P2I0, P2I8, and P2I32.

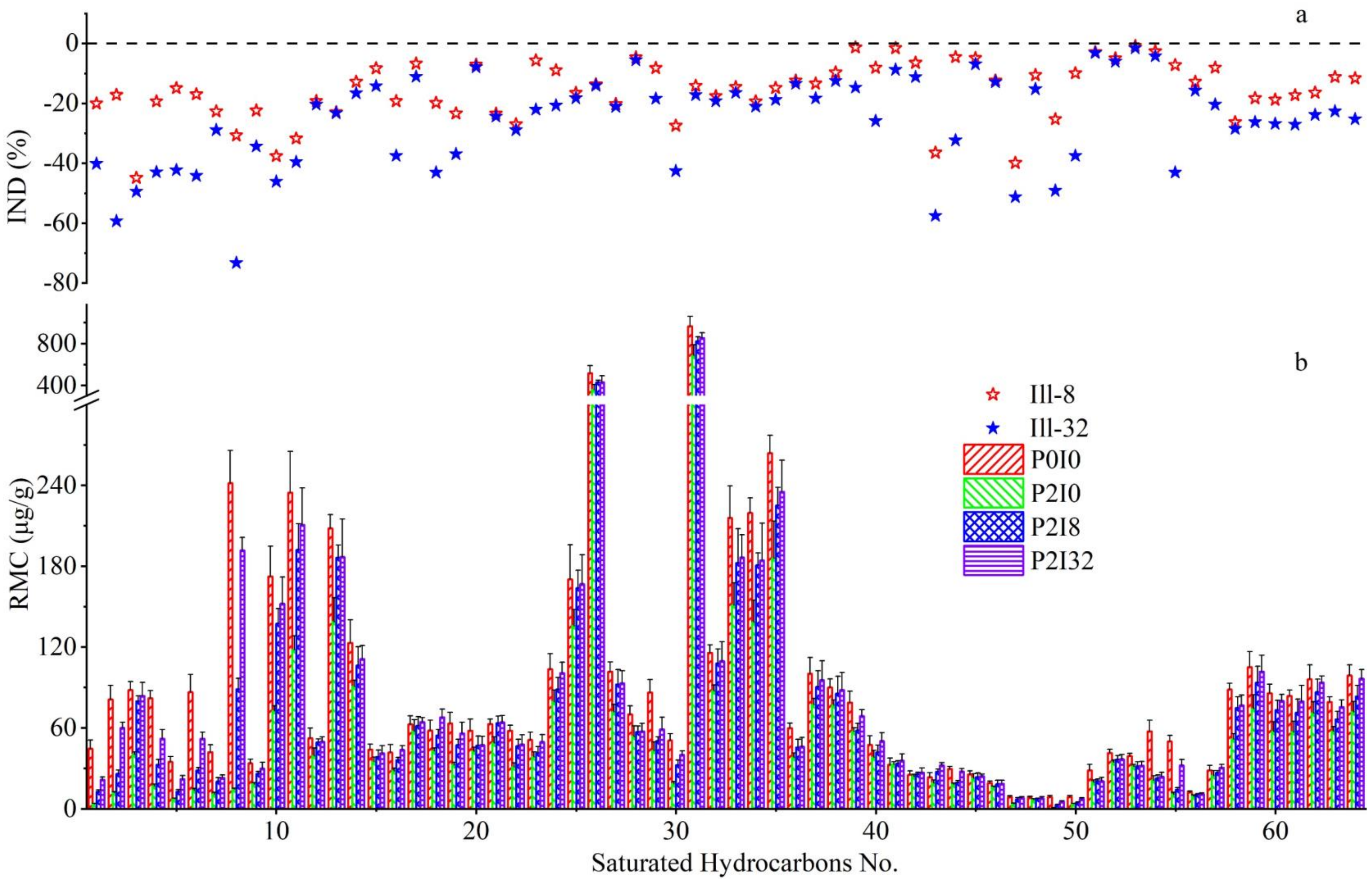

Figure S5. *IND* of illite on biodegradation of SHs (**a**) and *RMC* of SHs (**b**) in the illite-*Pst*HO. Note: See Table 1 for *IND*, *RMC* Ill-8, and Ill-32; see Table 2 for P0I0, P2I0, P2I8, and P2I32; see Table S2 for SHs No.

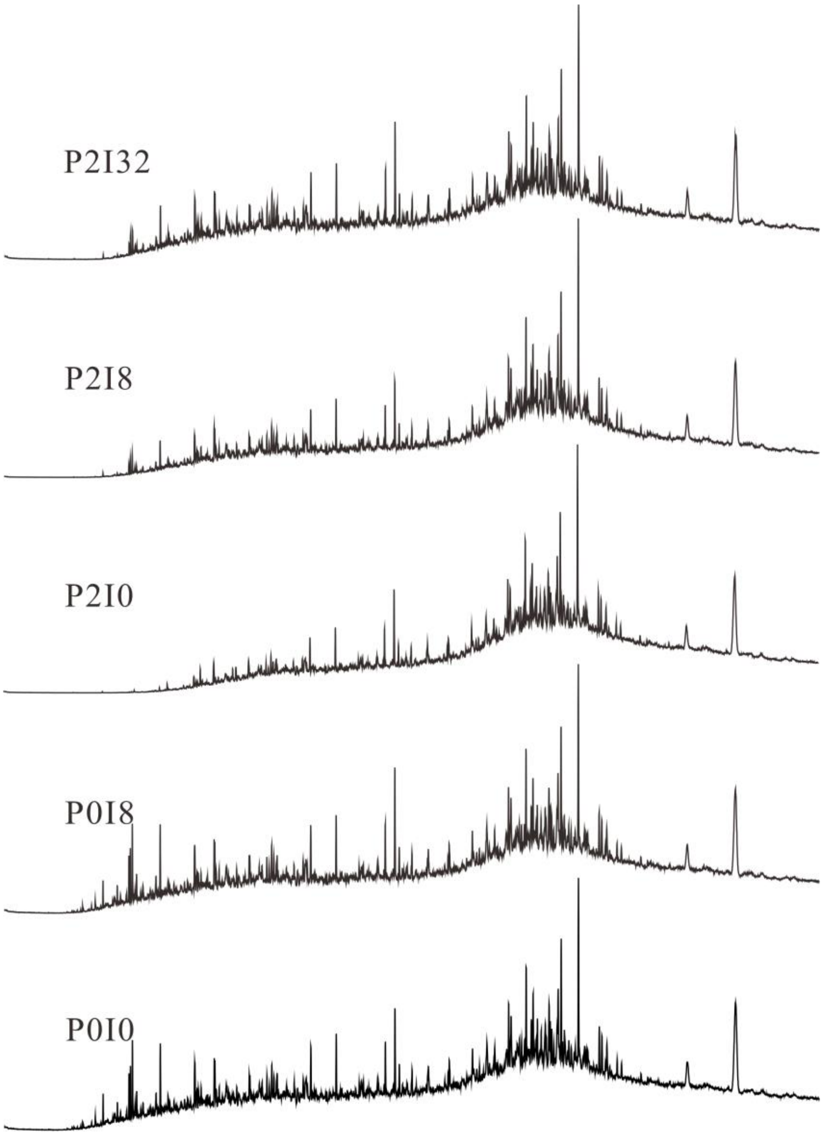

Figure S6. Total ion current diagrams of SHs in illite-*Pst*HO after 56 days at 35 °C. Note: Abbreviations are same as Table 2.

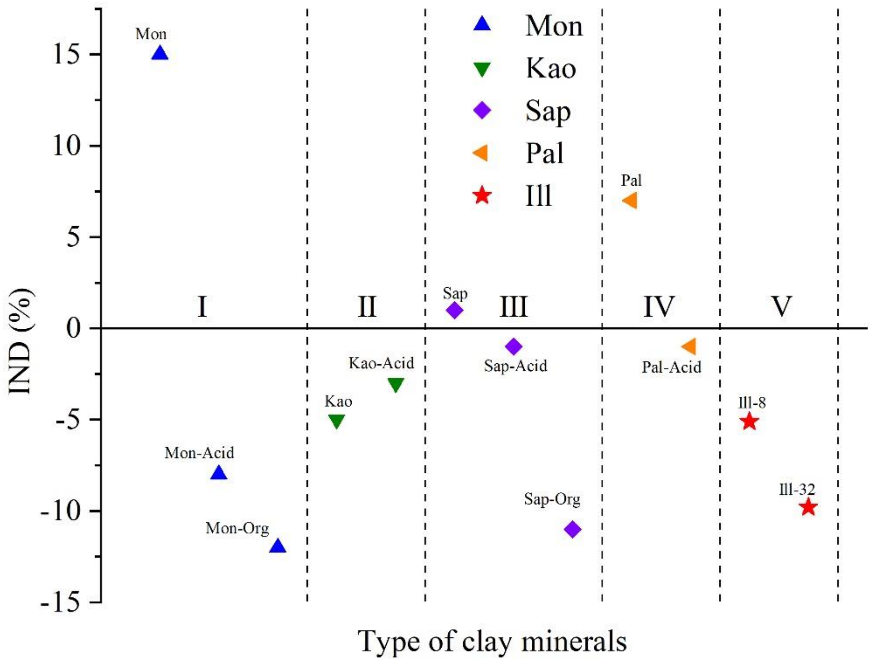

Figure S7. *IND* of clay minerals on the biodegradation of SHs. Note: Areas I, II, III, IV, and V represent Mon, Kao, Sap, Pal, and illite respectively; Ver denotes vermiculite; see Table 1 for the abbreviations; data in this figure are derived from the present study and the literature [25].

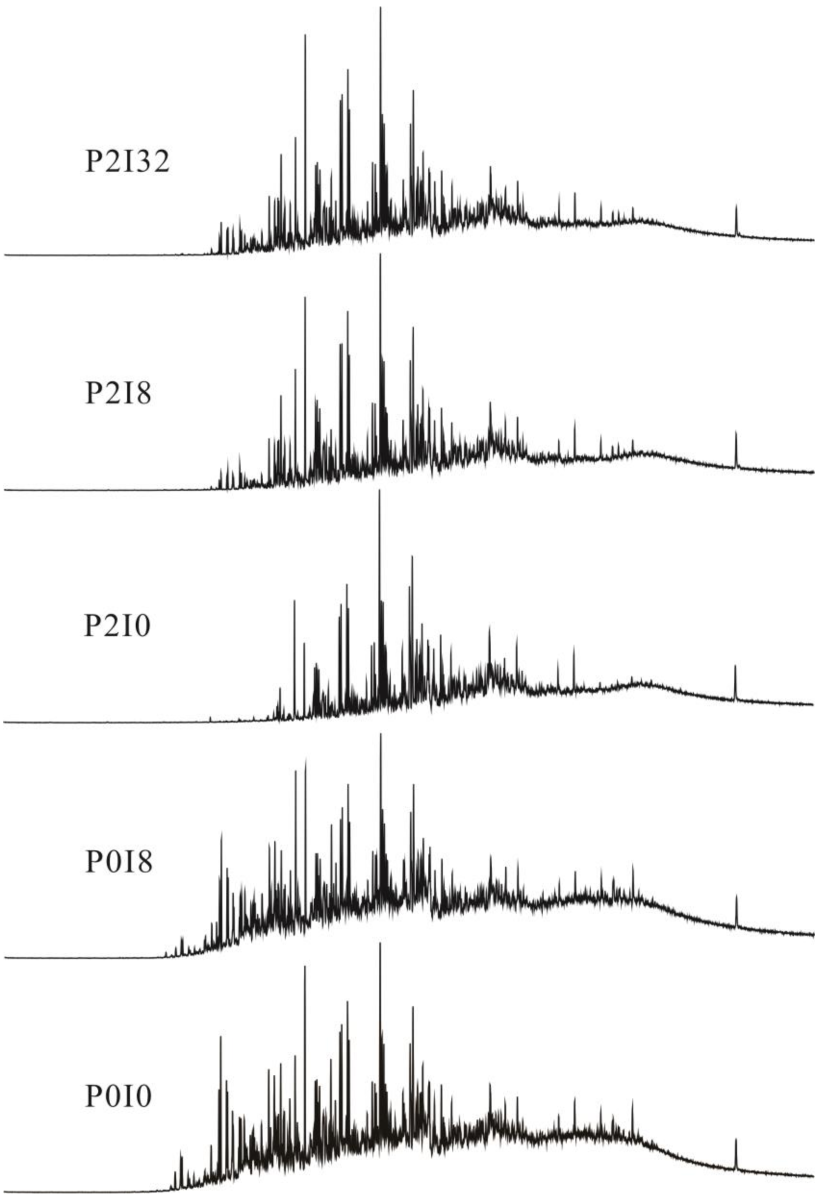

Figure S8. Total ion current diagrams of AHs in the illite-*Pst*HO after 56 days at 35 ℃
Note: Abbreviations are same as Table 2.

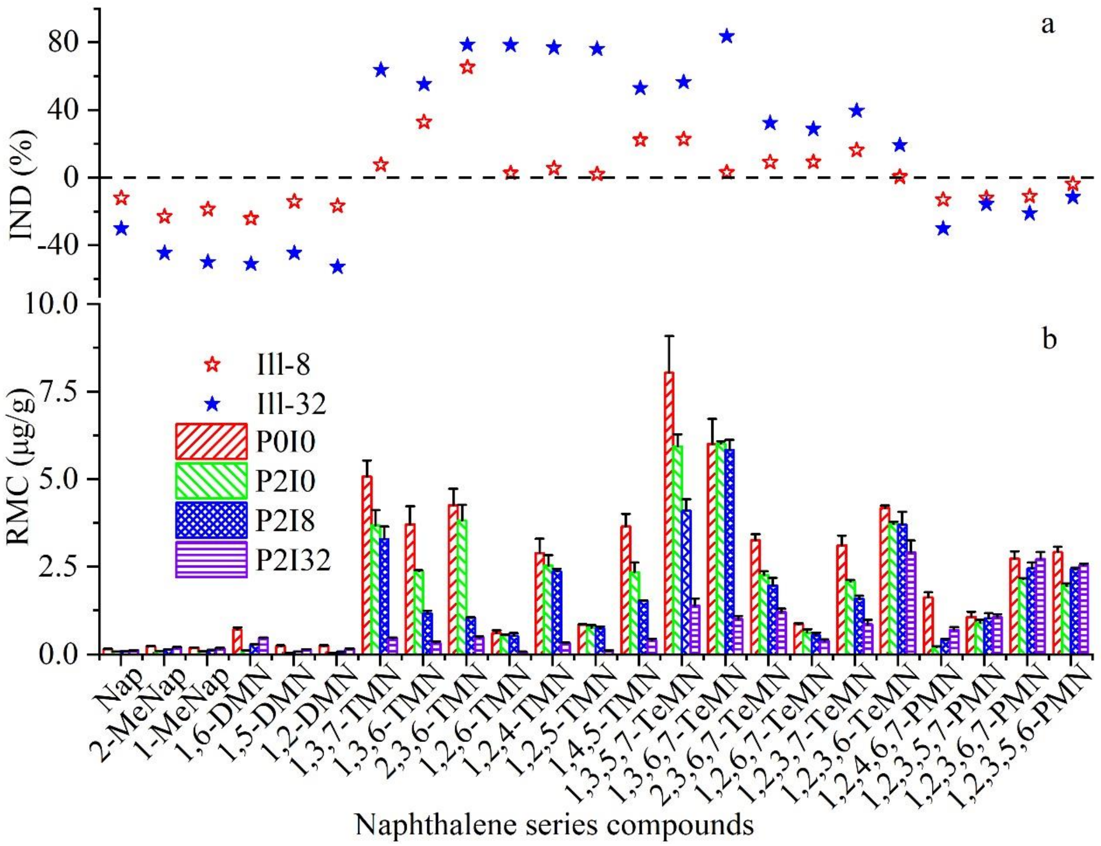

Figure S9. *IND* of illite on the biodegradation of naphthalene series compounds (**a**) and *RMC* of naphthalene series compounds (**b**) in the illite-*Pst*HO. Note: See Table 1 for *IND*, *RMC*, Ill-8, and Ill-32; see Table 2 for P0I0, P2I0, P2I8, and P2I32; see Table S3 for abbreviations of naphthalene series compounds.

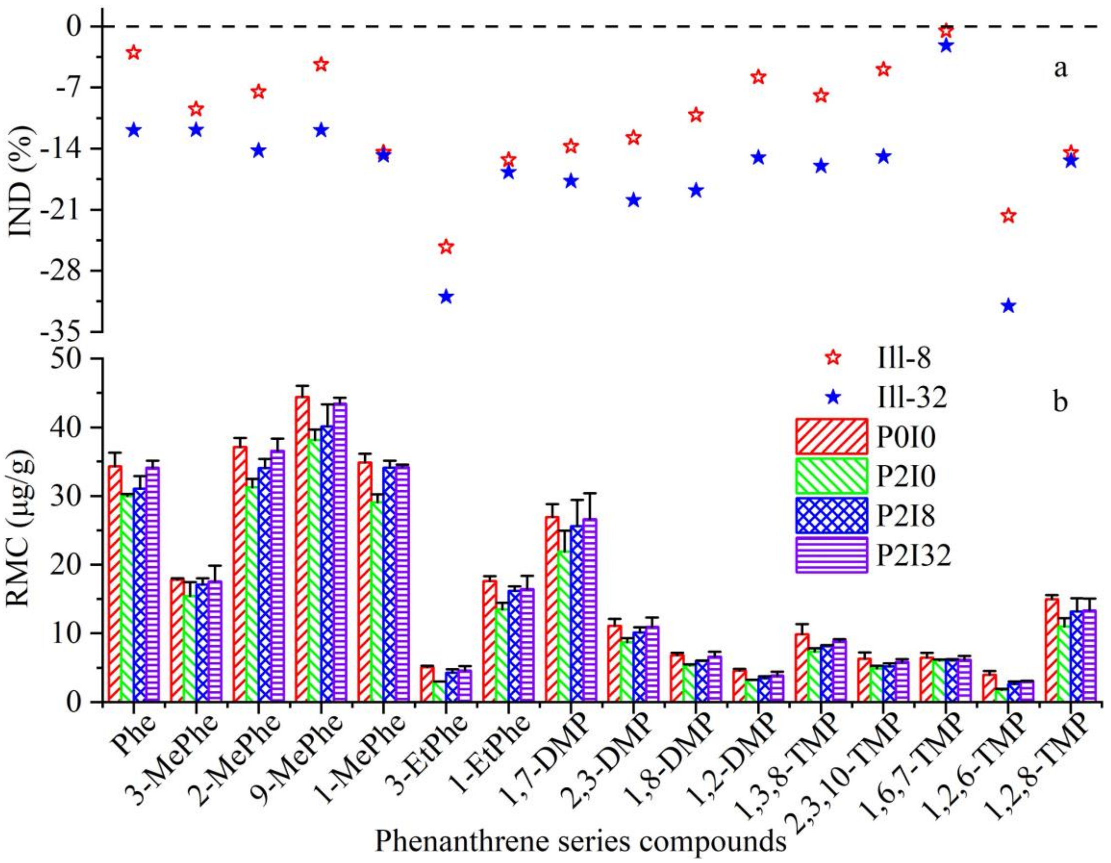

Figure S10. *IND* of illite on the biodegradation of phenanthrene series compounds (**a**) and *RMC* of phenanthrene series compounds (**b**) in the illite-*Pst*HO. Note: See Table 1 for *IND*, *RMC*, Ill-8, and Ill-32; see Table 2 for P0I0, P2I0, P2I8, and P2I32; see Table S3 for abbreviations of phenanthrene series compounds.

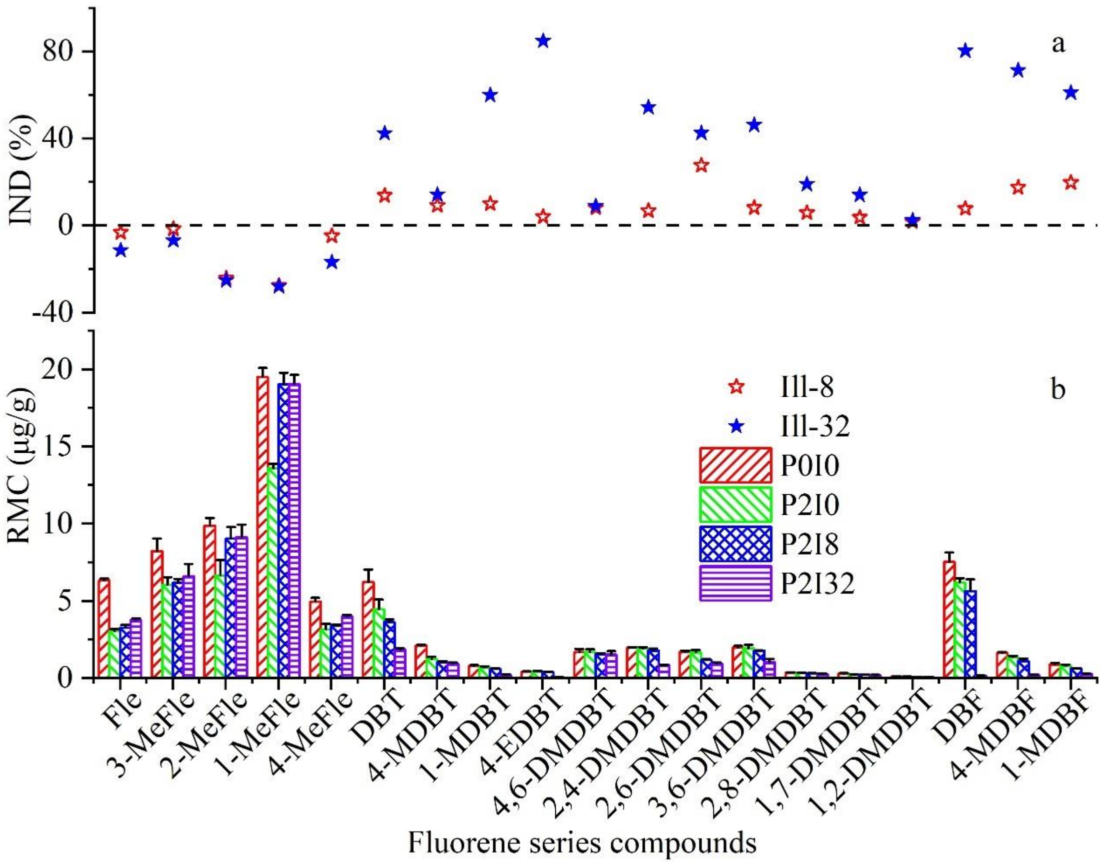

Figure S11. *IND* of illite on the biodegradation of fluorene series compounds (**a**) and *RMC* of fluorene series compounds (**b**) in the illite-*Pst*HO. Note: See Table 1 for *IND*, *RMC*, Ill-8, and Ill-32; see Table 2 for P0I0, P2I0, P2I8, and P2I32; see Table S3 for abbreviations of fluorene series compounds.

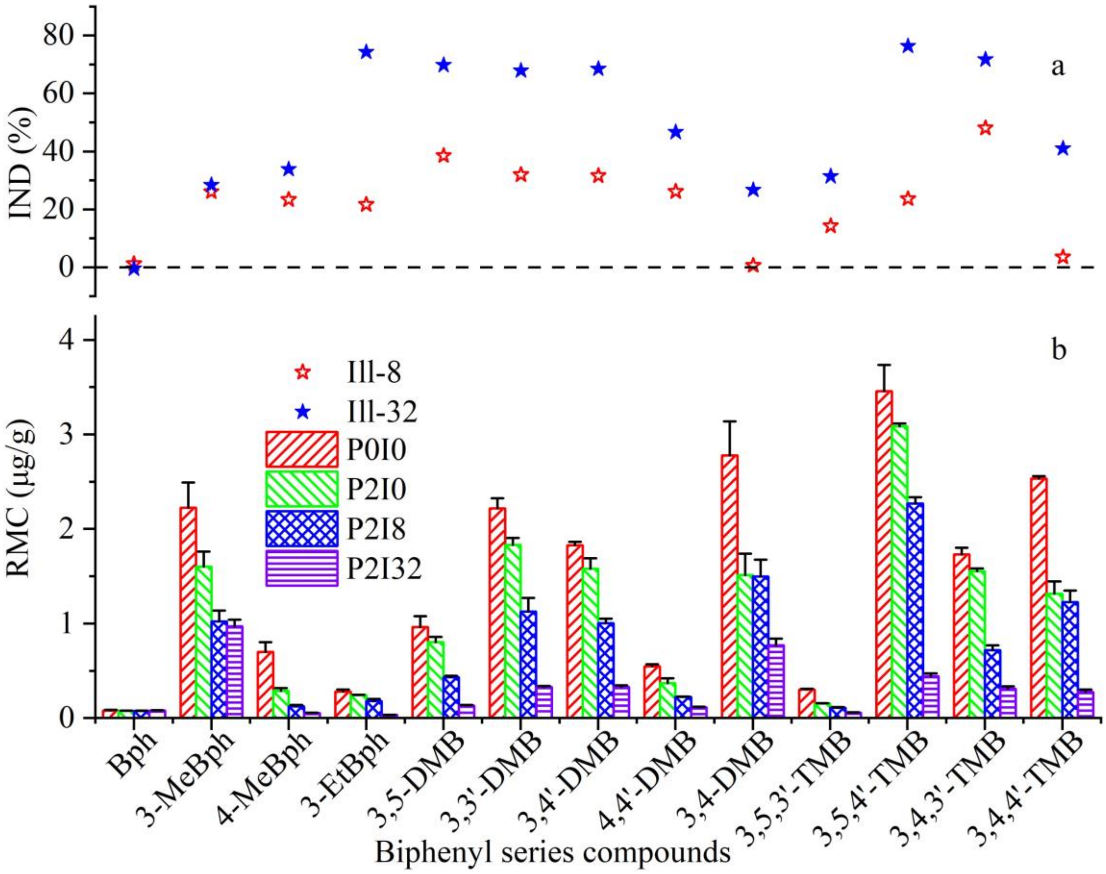

Figure S12. *IND* of illite on biodegradation of biphenyl series compounds (**a**) and *RMC* of biphenyl series compounds (**b**) in the illite-*Pst*HO. Note: See Table 1 for *IND*, *RMC*, Ill-8, and Ill-32; see Table 2 for P0I0, P2I0, P2I8, and P2I32; see Table S3 for abbreviations of biphenyl series compounds.

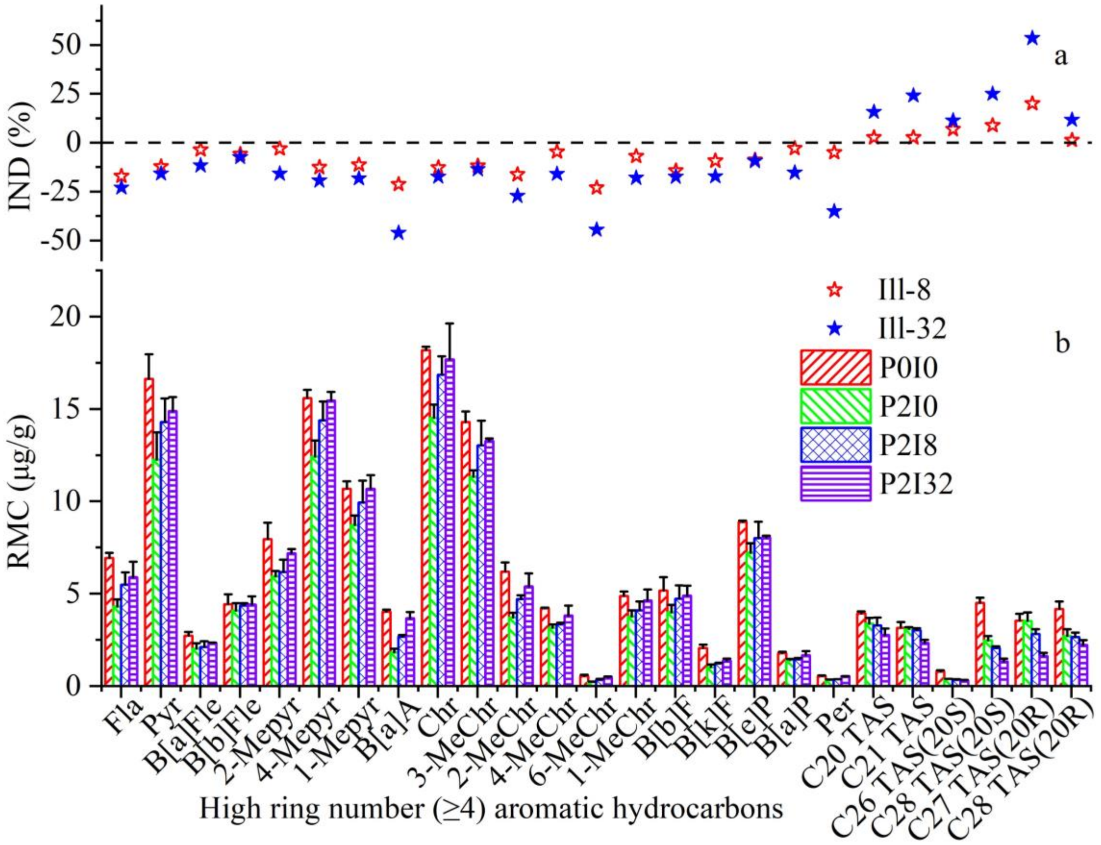

Figure S13. *IND* of illite on the biodegradation of HRAHs (**a**) and *RMC* of HRAHs (**b**) in the illite-*Pst*HO. Note: See Table 1 for *IND*, *RMC*, Ill-8, and Ill-32; see Table 2 for P0I0, P2I0, P2I8, and P2I32; see Table S3 for abbreviations of HRAHs series compounds.

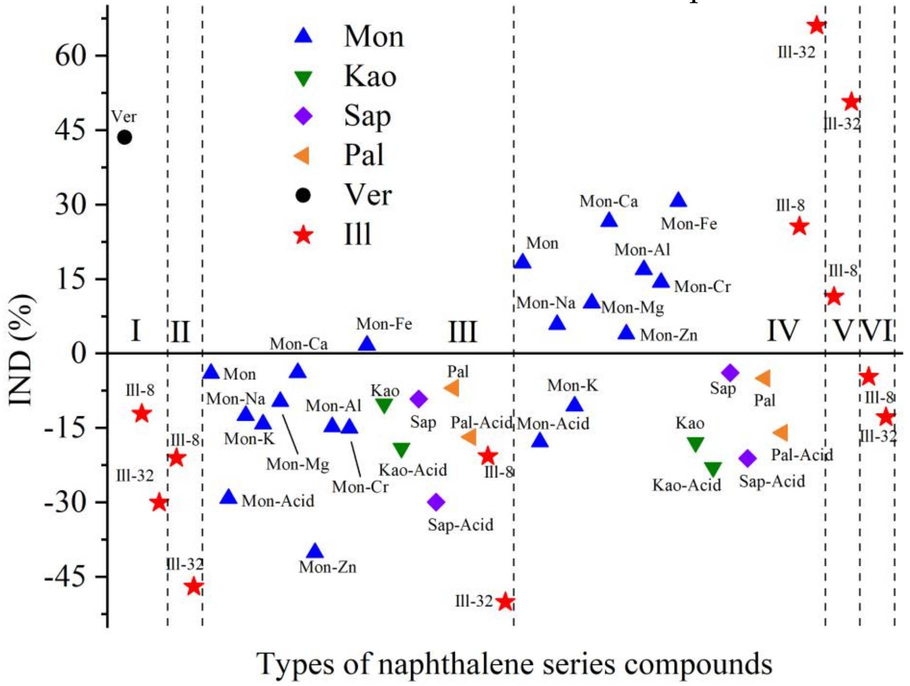

Figure S14. *IND* of clay minerals on the biodegradation of naphthalene series compounds. Note: Areas I, II, III, IV, V and VI represent Nap, MeNap, DMN, TMN, TeMN, and PMN respectively; see Table 1 and Table S3 for abbreviations； data in this figure are derived from the present study and the literature [23,30,36].

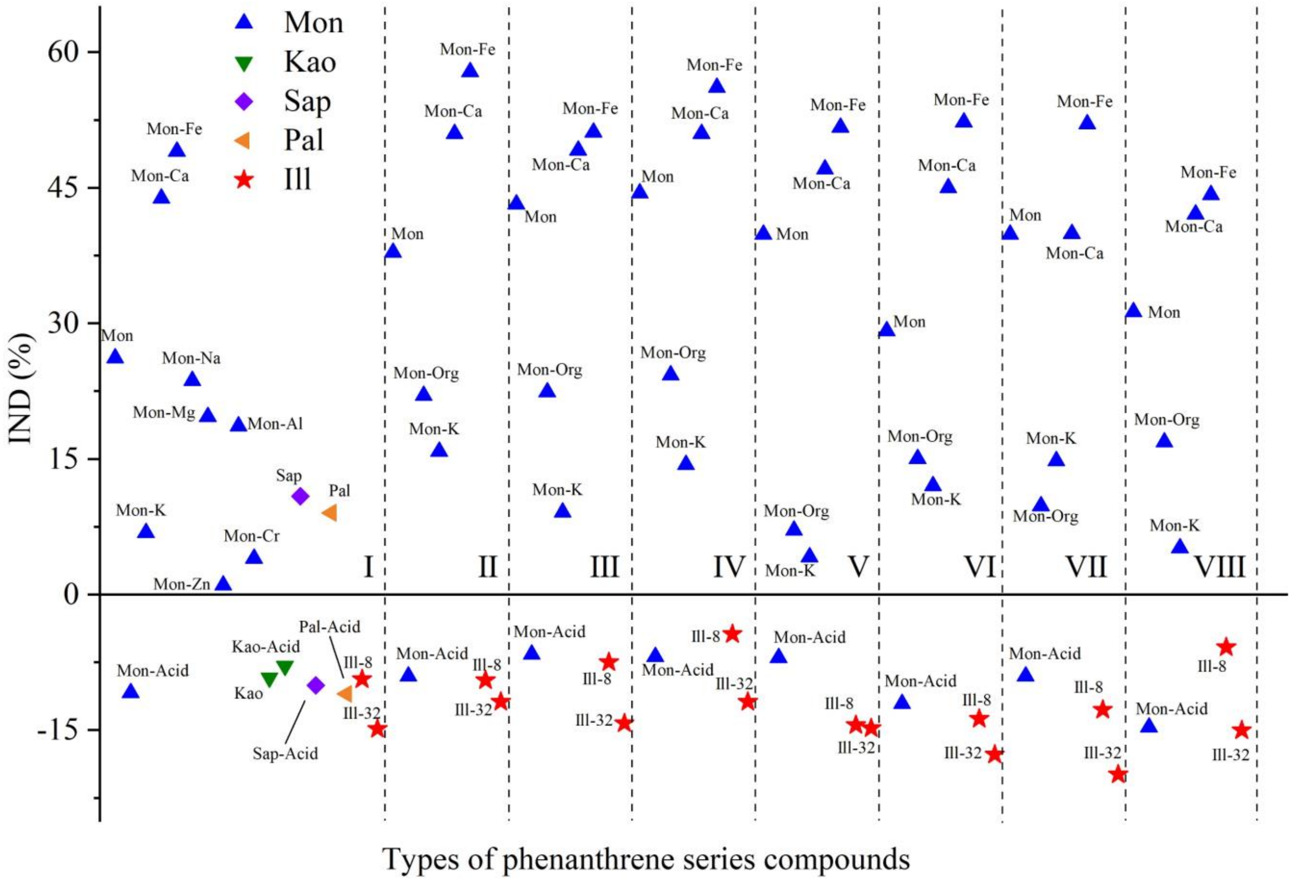

Figure S15. *IND* of clay minerals on the biodegradation of phenanthrene series compounds
Note: Area I represent the total amount of Phe, 3-MePhe, 2-MePhe, 9-MePhe, 1-MePhe, 3-EtPhe, 1-EtPhe, 1,7-DMP, 2,3-DMP, 1,8-DMP, and 1,2-DMP; areas II, III, IV, V, VI, VII, and VIII represent 3-MePhe, 2-MePhe, 9-MePhe, 1-MePhe, 1,7-DMP, 2,3-DMP, and 1,2-DMP, respectively; see Table 1 and Table S3 for abbreviations; data in this figure are derived from the present study and the literature [29,30,36].

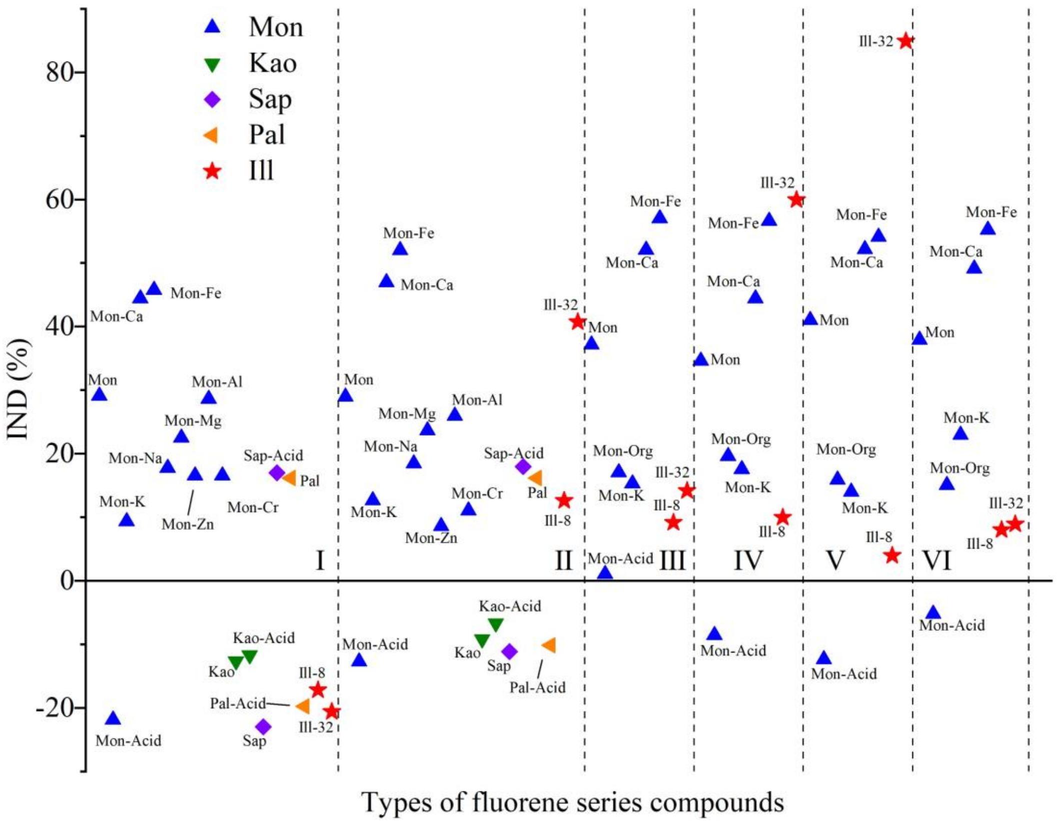

Figure S16. *IND* of clay minerals on the biodegradation of fluorene series compounds

Note: Area I represent the total amount of Fle and MeFle; area II represent the total amount of DBT, MDBT, and DMDBT; area III, IV, V, and VI represent 4-MDBT, 1-MDBT, 4-EDBT, and 4,6-DMDBT, respectively; see Table 1 and Table S3 for abbreviations; data in this figure are derived from the present study and the literature [29,30,36].

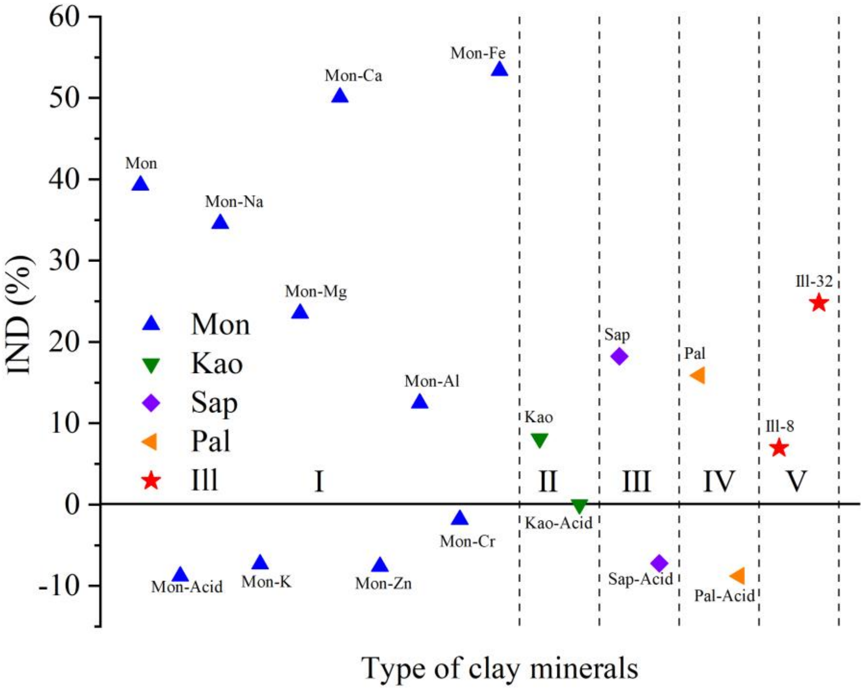

Figure S17. *IND* of clay minerals on the biodegradation of TAS. Note: See Figure. S8 for areas I, II, III, IV, and V; see Table 1 and Table S3 for abbreviations; data in this figure are derived from the present study and the literature [30,36].
